# Reticulocyte Hemoglobin as a Screening Test for Iron Deficiency Anemia: A New Cut-Off

**DOI:** 10.3390/hematolrep15010021

**Published:** 2023-03-14

**Authors:** Abdullah I. Aedh, Mohamed S. M. Khalil, Alaa S. Abd-Elkader, Mohamed M. El-Khawanky, Hamdan M. Alshehri, Amr Hussein, Ali A. Lafi Alghamdi, Abdulkarim Hasan

**Affiliations:** 1 Internal Medicine Department, College of Medicine, Najran University, Najran 55461, Saudi Arabia; aayahya@nu.edu.sa (A.I.A.);; 2 Clinical Pathology Department, Faculty of Medicine, Assiut University, Assiut 71515, Egypt; 3 Clinical Hematopathology, College of Medicine, Najran University, Najran 55461, Saudi Arabia; 4 Internal Medicine Department, Faculty of Medicine, Al-Azhar University, Cairo 11884, Egypt; 5 Laboratory Department, Prince Mishari bin Saud Hospital, Saudi Ministry of Health, Baljurashi 22888, Saudi Arabia; 6 Pathology Department, Faculty of Medicine, Al-Azhar University, Cairo 11884, Egypt

**Keywords:** Ret-Hb, iron deficiency, anemia, reticulocyte, latent iron deficiency

## Abstract

Introduction: Latent iron deficiency (LID), in which iron stores in the body are depleted without incidental anemia, poses a key diagnostic challenge. Reticulocyte hemoglobin content (Ret-Hb) is directly correlated with the functionally available iron for heme synthesis in erythroblasts. Consequently, Ret-Hb has been proposed as an efficient iron status marker. Aim: To assess the importance of Ret-Hb in detecting latent iron deficiency as well as its use in screening for iron deficiency anemia. Materials and Methods: A study involving 108 individuals was conducted at Najran University Hospital, 64 of whom had iron deficiency anemia (IDA) and 44 of whom had normal hemoglobin levels. All patients were subjected to complete blood count (CBC), reticulocyte percentage, Ret-Hb, serum iron, total iron binding capacity (TIBC), and serum ferritin measurements. Results: A significant decrease in Ret-Hb level was observed in IDA patients compared to non-anemic individuals, with a cut-off value of 21.2 pg (a value below which indicates IDA). Conclusion: The measurement of Ret-Hb, in addition to CBC parameters and indices, provides an accessible predictive marker for both iron deficiency (ID) and IDA. Lowering the Ret-Hb cut-off could better allow for its use as a screening parameter for IDA.

## 1. Introduction

Reticulocyte hemoglobin content (Ret-Hb) correlates directly with the functionally available iron for heme synthesis in erythroblasts. Consequently, Ret-Hb has been proposed as an efficient marker of iron status [1]. Several studies have suggested that a Ret-Hb measurement in peripheral blood samples is useful for the diagnosis of iron deficiency and the development of iron therapy response [2]. Latent iron deficiency (LID) is a diagnostic challenge in which body iron stores are deficient without incidental anemia. LID may go unrecognized for a long time and is suspected to be due to a decrease in serum ferritin levels [3,4]. It is important to identify cases of LID as most of them develop into iron deficiency anemia (IDA) if the iron condition is not corrected. In addition, individuals who suffer from LID usually complain of mysterious symptoms, including intense fatigue, epithelial cell devitalization (e.g., cheilitis), pica, hair loss, restless legs syndrome, thinner central cornea, decreased cognitive performance, behavioral disturbances, and enhanced osteoporosis in women [5,6,7,8]. In young people, there is evidence of an association between iron deficiency and cognitive function impairment. It is well known that cognition is crucial for quality of life (QoL) and encompasses various functions including attention, memory and concentration [4,9]. The exact mechanism by which IDA affects the brain is still not well understood; however, there are some supposed possibilities including abnormalities in neurotransmitter metabolism, alterations in brain energy metabolism and decreased myelin formation [4,10]. Leonard et al. in 2014 used an easy tool (IntegNeuro) to administer the assessment of cognitive function in young women and concluded that some cognitive change scores were significantly higher for ferritin improvers than non-improvers (irrespective of treatment group) and for women who had LID at the baseline and were treated with iron supplements [4]. LID is also known as “nonanemic iron deficiency” or “subclinical iron deficiency” where the transition from normal iron leveling to the state of IDA development entails two sequential processes including depletion, followed by exhaustion, of the iron storage compartment and the consequent depletion of the functional compartment. The development of IDA is a consequence of functional compartment depletion [11]. The mean intracellular hemoglobin content of the erythrocytes (MCH) is considered an inclusive measurement for both the availability of iron over the preceding 90–120 days and for the proper introduction of iron into intracellular hemoglobin [12]. By directly measuring the mean hemoglobin content (MHC) of the red blood cell precursors (reticulocytes), early stages of IDA may be identified at a time when other traditional biochemical parameters appear to be non-informative [13]. The measurement of ret-hemoglobin content is a known direct assessment of the iron incorporation into erythrocyte hemoglobin, so it is a direct estimate of the recent functional availability of such iron in the erythron [14]. A single biomarker is important to use for the diagnosis of IDA, but the use of a serum marker that can be easily identified as a screening marker is necessary [15,16]. Ret-He has already been suggested to be an additional marker for the screening of IDA [15]. Hence, in this study, we review the role of Ret-Hb in the diagnosis of LID and as a screening parameter for IDA.

## 2. Materials and Methods

### 2.1. Patients

The study involved 108 randomly selected individuals from the outpatient clinics of internal medicine at Najran University Hospital, Saudi Arabia, after obtaining the approval of the IRB ethics committee of Najran University and the informed consent of the participants. Patients with known hereditary hematological disorders or known hematological malignancies were excluded.

### 2.2. Sample Collection and Biochemical Analyses

Blood samples for CBC and Ret-Hb were collected in K3EDTA tubes and analyzed using an automated hematology analyzer (Sysmex XS 500i, Tokyo, Japan; Sysmex, https://www.sysmex.com/ (accessed on 1 October 2022). Serum biochemical analyses (iron and ferritin) were performed using a COBAS C311 (Roche, Basel, Switzerland (https://www.roche.com/ (accessed on 1 October 2022) automated chemical analyzer.

Patients with known hematological diseases or who were on a long-term drugs (especially chemotherapy or radiotherapy) were excluded to avoid ferritin variation. Pregnant women were also excluded.

### 2.3. Assessment of Anemia and Iron Deficiency

We classified anemia according to the WHO definition for anemia, as follows: Hb < 12.0 g/dL in females and Hb < 13.0 g/dL in males. Iron deficiency was defined as transferrin saturation (TSAT) < 20% and ferritin level in serum < 100 ng/mL, according to Muñoz [17]. A serum ferritin level of <30 ng/mL with normal Hb was considered to indicate insufficient iron store or iron deficiency. The endpoint of this research was the validation of Ret-Hb as a screening marker for LID and IDA in adults, considering its correlation with other parameters. Being overweight or obese can affect iron and ferritin levels as these conditions may leave the patient at risk of developing subclinical inflammation and certain chronic diseases or complications such as obstructive sleep apnea, ischemic heart disease, cor pulmonale, and many others. At the same time, they can increase the likelihood of iron deficiency and iron deficiency anemia. According to previous studies, there is an increasing trend in the prevalence of obese and overweight individuals in Saudi Arabia. We included participants suffering from obesity or being overweight after exclusion of the associated complications. We repeated testing of some samples twice or sent samples to another laboratory for assurance when applicable or in the case of unusual results. 

Cancer patients were excluded from this study as ferritin testing is a less useful or accurate tool in oncology patients, especially patients with solid tumors due to ferritin elevation in patients with different solid tumors and the association with more progressive diseases and shorter survival when elevated.

### 2.4. Current Cutoffs

Diagnosis of IDA and LID with the use of Ret-Hb is dependent on comparing patient results with supposed diagnostic cutoffs. However, Ret-Hb cutoffs currently recommended by several studies demonstrate considerable inconsistency. Between 25–29 pg cutoffs are used for the diagnosis of iron deficiency and around 21 pg are used for iron deficiency anemias. Herein we tested a cutoff in our institution. 

### 2.5. Statistical Analysis

Data were analyzed using the IBM SPSS 20 software. An independent sample *t*-test was performed for normally distributed variables, and the results are presented as mean ± standard deviation. 

Receiver operating characteristic (ROC) curve analysis was performed to identify the optimal Ret-Hb cutoff value for predicting IDA. The statistical significance was set at *p* < 0.05.

## 3. Results

No statistically significant difference was observed between anemic and non-anemic groups in terms of RC; otherwise, significant differences were seen in all other items, as shown in Table 1. 

Patients with IDA showed significantly (*p* < 0.05) decreased levels of hemoglobin, MCV, MCH, MCHC, serum iron, and ferritin, along with significant increases in RDW and platelet count, when compared with normal individuals of both sexes. However, these decreased amounts were still above the lower normal limit of Hb (12 g/dL in female and 13 g/dL in male patients) and the limit of MCV (80 for both sexes).

There was a highly significant (*p* = 0.0001) decrease in reticulated hemoglobin level in anemic patients of both sexes, in comparison to non-anemic patients, but the reticulocyte count did not show any significant difference.

In individuals with normal hemoglobin, Ret-Hb levels were significantly decreased in males with low ferritin levels and showed a tendency to decrease in females, but the reticulocyte count (RC) did not show any significant difference among groups in both sexes.

The low ferritin level group showed a significant increase in UIBC level and significantly lower hemoglobin levels in both sexes.

MCV and MCH decreased significantly in normal males with low ferritin levels, and there was a tendency towards decreased levels in females of the same group (Table 2).

Male IDA patients showed significant decreases in Ret-Hb, RBCs, Hb, MCHC, and serum iron levels, but showed no significant difference (*p* > 0.05) in MCV, MCH, RDW, PLT, UIBC, ferritin, and RC, when compared to normal Hb individuals with low ferritin levels.

Female IDA patients showed significant decreases in the levels of Ret-Hb, RBCs, Hb, MCV, MCH, MCHC, serum iron, and ferritin, as well as significant increases in RDW, PLT, and UIBC, but without any significant difference in RC, when compared to the other groups (Table 3).

Reticulocyte hemoglobin showed positive correlations with hemoglobin level (*p* = 0.0001, r = 0.819) and serum ferritin levels (*p* = 0.0001, r = 0.540) and tended to correlate with serum iron levels (*p* = 0.065, r = 0.232; Table 4). 

We observed a Ret-Hb cutoff value of 21.2 pg (100.0% sensitivity, 64.1% specificity), values below which can predict IDA. We considered 100% sensitivity to apply this parameter as a screening value for IDA.

**Figure 1:**
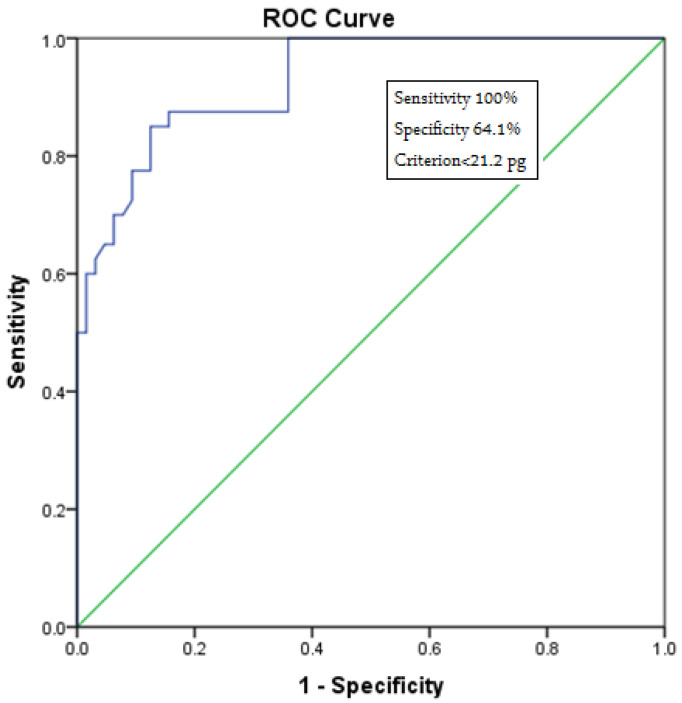
Receiver operating characteristic (ROC) curve analysis for reticulocyte hemoglobin levels in normal individuals and patients with IDA. This figure indicates the cutoff value for Ret-Hb, the lower value of which suggests IDA (cutoff = 21.2 pg) with 100% sensitivity.

The results of the receiver operating characteristic (ROC) analysis for reticulocyte hemoglobin levels in normal individuals and patients with IDA are shown in Figure 1.

Regarding samples that were repeated either in the sample laboratory or a different one, all results were compatible with the first results from our institution. 

## 4. Discussion

Iron deficiency anemia may be detected relatively late when considering classic laboratory parameters such as Hb, mean corpuscular volume, and mean corpuscular hemoglobin. A single biomarker to detect either IDA or LID is rarely used; however, markers that can be easily used for screening are necessary and are still required for further research worldwide [7,18]. The lifespan of circulating erythrocytes is approximately 120 days. Changes in Hb and MCV values usually occur at a later time point, when the IDA is already fulminant [14]. In CBC, the MCV was measured at under 80 fL, but its normal value is between 80 to 100 fL. This microcytic anemia can be observed in anemia related to chronic disease, thalassemia, sideroblastic anemia, and mainly in chronic iron deficient anemia. Microcytic cells in the setting of iron deficient anemia may appear to have larger areas of central pallor [18]. Anemia can be a consequence of absolute iron deficiency which is due to chronic blood loss; however, in many patients of chronic diseases, enhanced formation of pro-inflammatory cytokines particularly interleukin 1 (IL-1), interferon gamma (IFN-γ) or tumor necrosis factor alpha (TNF-α) leads to the development of functional IDA and anemia of chronic disease. These pro-inflammatory types of cytokines suppress the production of renal erythropoietin, but also directly inhibit bone marrow erythropoiesis [19,20]. The key diagnostic parameters routinely used for β- and α-thalassemia are ferritin and hemoglobin analysis (HbA_2_ and Hb abnormality) in addition to DNA analysis; however, several simple screening indices are highly recommended in endemic areas to differentiate between IDA and thalassemia traits, and recently these were encouraged along with other potentially better performing indices [21]. The sideroblastic anemias are a group of acquired and inherited bone marrow disorders defined as a pathological iron accumulation in erythroid precursor mitochondria. The abnormal, iron-laden mitochondria are seen encircling erythroblast nuclei, giving rise to characteristic morphological features of the sideroblastic anemias. The ring sideroblast was originally recognized in the 1940s and codified as a class of anemias in the 1960s. Similarly to most hematological diseases, understanding the molecular genetic basis of anemias is important for better understanding of their pathogenesis and to face the diagnostic and therapeutic challenges presented by them [22].

Reticulocytes are formed in the bone marrow and then develop into mature erythrocytes two days later, at which point they are seen in the peripheral blood [13]. Therefore, using blood samples to determine Hb content in reticulocytes is helpful to analyze and assess iron levels via reticulocytes [23]. The assessment of hemoglobin content in reticulocytes can accurately reflect iron levels [24].

Our findings demonstrated that the levels of Ret-Hb in IDA were decreased significantly (*p* = 0.000), in comparison with healthy individuals of both sexes, thus establishing that low Ret-Hb is a good indicator of iron deficiency anemia, which is consistent with many previous studies [25,26,27]. 

The importance of incorporating Ret-Hb into the diagnostic or screening panel of iron deficiency is due to the bioavailability of iron in the synthesis of hemoglobin in newly formed red blood cells (reticulocytes), because Ret-Hb levels are not as influenced by other conditions (e.g., inflammation) as those of acute phase reactants such as ferritin and transferrin [28], or the fluctuations caused by the diurnal variation and/or the quality of food intake, unlike serum iron [29,30]. In addition, the Ret-Hb under B anemias is similar [31,32], whereas Ret-Hb deficiency and high or normal levels in thalassemia have been observed, as the latter coincides with an increase in iron levels as a result of multiple blood transfusions or enhanced iron absorption secondary to ineffective erythropoiesis and hepcidin suppression [33,34]. 

Our results indicated significant decreases in Ret-Hb (*p* = 0.018), MCV, and MCH in males with low ferritin but normal hemoglobin concentrations, whereas females presented a non-significant decrease in mean Ret-Hb level, when compared to normal individuals. Therefore, we consider Ret-Hb to be a good indicator of decreased iron levels in males in general, especially in the presence of hypochromia and microcytosis. In contrast, when measuring Ret-Hb in women, we did not observe a significant difference between women with low and normal ferritin levels. 

The most common cause of LID is menstrual blood loss among women of reproductive age (mean age of 30.2 ± 10 years) [35]. Women of reproductive age have two different periods per month: (a) the menstrual period, in which blood loss occurs causing iron store depletion. During non-menstrual days (b), the female compensates for the deficiency of iron by increasing the reticulocyte hemoglobin as a reaction to blood loss and not only to an absolute iron deficiency, despite the joint presence of the two conditions. This can be demonstrated by the relatively high percentage of reticulocytes with normal hemoglobin content in females of reproductive age with low levels of ferritin.

Our study emphasized the importance of reticulocyte hemoglobin content (Ret-Hb) as a predictive marker of iron deficiency anemia, and as an early indicator of iron deficiency without the incidence of anemia, thus emphasizing the importance of using Ret-Hb measurement as a screening test for iron deficiency. We observed a Ret-Hb cutoff value of 21.2 pg (100.0% sensitivity, 64.1% specificity), values below which can predict IDA. We considered 100% sensitivity to apply this parameter as a screening value for IDA. Another study has stated that the cut-off value of Ret-Hb is 29.3 pg (90.6% sensitivity, 66.7% specificity) in female patients with IDA [36]. This difference in the cut-off value might be due to the difference in the degree of sensitivity, as we used 100% sensitivity to apply the Ret-Hb measurement as a screening test, as opposed to 90.6% in the previous study. Uçar et al. used a Ret-Hb cut-off value of <90% with a sensitivity of 49.1% [37]. Toki et al. used a Ret-Hb cut-off value of 28.5 pg and had a specificity of >90% and 68% sensitivity. With a higher cut-off (30.9 pg), the sensitivity increased to 92%, whereas the specificity dropped back to 81% for the diagnosis of iron deficiency [38]. For LID, Tiwari et al. assessed the diagnostic usefulness of Ret-Hb in blood donors with respect to sTfR and revealed that with a cut-off value < 28 pg, a higher sensitivity (92%) and specificity (97%) were reported [39].

Ferritin can play a role when C-reactive protein and IL-6 (a critical cytokine)act as pro-inflammatory cytokines by mediating fever and the acute inflammatory response to lower the fever and critical cytokine levels, which in turn reduces the hyperinflammatory response [40,41]. In most laboratories currently, the serum ferritin level is used to indicate the body’s iron store as it is a non-invasive method that provides reliable results, near to the invasive gold-standard method for body iron store; however, the difference in cut-off of ferritin levels to define depleted iron stores can also be seen [24,41].

The stores of ferritin in the human body are predominantly found in the macrophages of the reticuloendothelial system and in hepatocytes. Macrophages phagocytose damaged or aged erythrocytes by recycling the iron contained in heme using heme oxygenase-1 to release the iron; this recycling accounts for about 90% of the body’s daily needs from iron, with only around 10% being met by intestinal absorption [42]. Iron is released from those storage sites as ferritin (II) via ferroportin in the cell membrane, then reoxidation of ferritin (II) to ferritin (III) by the ferroxidase enzyme ceruloplasmin occurs, followed by the loading of ferritin (III) onto transferrin for its systemic distribution to other sites [43]. Transferrin saturation is a marker for testing the amount of iron available for the process of erythropoiesis or other cellular requirements [44].

Serum ferritin level testing is widely used for the detection of IDA and LID; however, certain physical and demographic characteristics alter the iron homeostasis process and affect serum ferritin levels. These conditions include old age, obesity, and specific inflammatory conditions such as congestive heart failure. Patients who are overweight or obese have increased levels of hepcidin, likely due to adiposity-related inflammation, resulting in restricted dietary iron absorption and reduced transferrin saturation levels [45,46]. Serum ferritin levels are higher in some chronic inflammatory diseases even in non-obese individuals or in cases of low-grade inflammation [44,47]. Inadequate iron for erythropoiesis, as mainly determined by bone marrow aspiration and is frequently found in elderly people, even those with serum ferritin levels up to 75 μg/L [48,49]. Striking increases in serum ferritin levels can also occur in infectious events and in cases of acute inflammatory process [44]. Identifying accurate parameters to detect LID is always of interest and non-invasive tools are preferred, which raises the importance of hemoglobin tests such as Ret-Hb. New hematology analyzers can measure the hemoglobin content within the reticulocytes through the principles of fluorescence flow cytometry combined with reportable/diagnostic parameters to provide valuable diagnostic information. Such information is evaluated internally in the laboratory to check the results and to complete the diagnosis findings [24,48].

We excluded pregnant women from this study, as it is supposed that describing the iron status of pregnant women is often challenging in similar research due to the limited nature of available data and the different cutoffs, diagnostic criteria and pathophysiology of anemia. During pregnancy, a marked physiologic increase in demand for absorbed iron is present to expand the red blood cell mass of the woman and to secure the required iron supply for the placental function needed to grow the fetus and to complete a normal pregnancy without developing iron deficiency or IDA or taking iron supplements, the woman should have iron stores in her body at conception of ≥500 mg, which corresponds to serum ferritin concentrations of 70–80 μg/L [49,50,51].

The limitations of the present study include the low number of studied patients (as the study was performed in a single institutional center) and the exclusion of pregnant women and patients with some chronic diseases. In addition, we did not evaluate other factors which might be affected in the context of IDA or LID, such as soluble transferrin receptor, transferrin, and sTfR/lF index. As such, these and similar factors should be taken into consideration in future research.

## 5. Conclusions

The measurement of Ret-Hb content can provide an early indication of iron deficiency, thus it could serve as a screening test for the primary diagnosis of IDA. We observed a Ret-Hb cutoff value of 21.2 pg (100.0% sensitivity, 64.1% specificity), values below which can predict IDA. We considered 100% sensitivity to apply this parameter as a screening value for IDA. Further sensitive and powerful parameters for the early detection of iron deficiency anemia are still required.

## Figures and Tables

**Table 1:** Clinical parameters of normal individuals and anemic patients by gender.

Parameter	Hemoglobin Level	Normal Hemoglobin (n = 23) and Anemic (n = 12), Males			Normal Hemoglobin (n = 21) and Anemic (n = 52), Females		
		Mean	Std. Deviation	*p* Value	Mean	Std. Deviation	*p* Value
Age	Normal Hb	27.5	17.8	0.342	34.2	16.3	0.298
	Low Hb (anemia)	31.5	19.7		29.3	10.7	
Hb (g/dL)	Normal Hb	15.7	1.3	0.000	12.8	0.6	0.000
	Low Hb (anemia)	10.6	1.1		9.3	1.4	
MCV	Normal Hb	83.4	6.3	0.000	80.1	6.0	0.000
	Low Hb (anemia)	71.2	6.3		68.1	7.5	
MCH	Normal Hb	27.2	2.6	0.000	25.3	2.5	0.000
	Low Hb (anemia)	21.5	2.2		19.9	3.2	
MCHC	Normal Hb	32.6	1.0	0.000	31.6	1.2	0.000
	Low Hb (anemia)	30.2	1.8		29.0	1.9	
RDW	Normal Hb	13.6	1.5	0.000	14.5	1.9	0.000
	Low Hb (anemia)	16.3	2.0		19.2	3.1	
PLT	Normal Hb	295.5	63.3	0.006	297.3	77.4	0.016
	Low Hb (anemia)	416.8	159.1		361.9	109.4	
Iron	Normal Hb	106.6	47.3	0.000	57.5	34.6	0.000
	Low Hb (anemia)	26.8	12.2		28.7	15.8	
Ferritin	Normal Hb	134.1	67.4	0.000	43.1	51.6	0.000
	Low Hb (anemia)	13.3	10.1		6.3	5.2	
RC	Normal Hb	1.3	0.4	0.472	1.1	0.3	0.328
	Low Hb (anemia)	1.4	0.3		1.2	0.4	
Ret-Hb	Normal Hb	30.8	3.3	0.000	26.5	3.7	0.000
	Low Hb (anemia)	21.7	2.1		19.1	4.5	

MCV, mean corpuscular volume; MCH, mean corpuscular hemoglobin; PLT, platelets; RDW, red cell distribution width.

**Table 2:** Clinical data of normal hemoglobin individuals classified based on ferritin level and gender.

Parameter	Ferritin Levels	Normal Ferritin (n = 17) and Low ferritin (n = 6) in Males			Normal Ferritin (n = 8) and Low Ferritin (n = 13) Females		
		Mean	SD	*p* Value	Mean	SD	*p* Value
Age	Normal	27.5	17.8	0.562	34.2	16.3	0.114
	Low	27.0	11.0		30.2	10.0	
RBC	Normal	5.8	0.4	0.965	5.269	0.5573	0.246
	Low	5.8	0.6		4.989	0.4969	
Hb	Normal	15.9	1.1	0.005	13.138	0.4984	0.037
	Low	13.3	0.4		12.577	0.5862	
MCV	Normal	84.7	5.2	0.004	81.163	6.9943	0.258
	Low	72.1	1.9		78.262	5.1906	
MCH	Normal	27.7	2.2	0.010	25.200	3.0393	0.879
	Low	23.1	1.6		24.377	2.2260	
MCHC	Normal	32.6	1.0	0.399	32.187	1.2218	0.057
	Low	32.0	1.4		31.208	0.9836	
RDW	Normal	13.4	1.3	0.096	14.000	2.0543	0.348
	Low	15.3	2.6		14.823	1.8065	
PLT	Normal	282.0	50.3	0.003	315.500	64.2206	0.413
	Low	410.0	48.1		286.154	85.0489	
Iron	Normal	112.7	45.8	0.098	52.809	40.0354	0.637
	Low	54.2	22.8		60.396	32.1350	
UIBC	Normal	218.8	69.6	0.001	261.363	51.9098	0.029
	Low	521.4	331.8		353.008	101.5408	
Ferritin	Normal	147.7	57.1	0.006	91.800	56.0003	0.000
	Low	19.2	5.9		13.069	6.6928	
RC	Normal	1.4	0.4	0.120	1.079	0.3778	0.970
	Low	0.8	0.4		1.085	0.3141	
Ret-Hb	Normal	31.3	2.9	0.018	27.146	2.9125	0.303
	Low	25.8	1.5		25.412	4.6323	

**Table 3:** Clinical data of normal Hb individuals with low iron stores and patients with IDA by gender.

		Normal Hb Low Ferritin Group (n = 6) and IDA Patients (n = 12) in Males			Normal Hb Low Ferritin Group (n = 13) and IDA Patients (n = 52) in Females		
		Mean	Std. Deviation	*p* Value	Mean	Std. Deviation	*p* Value
Age	Normal Hb low Ferritin	27.0	11.0	0.164	30.2	10.0	0.775
	IDA	31.5	19.7		29.3	10.7	
RBC	Normal Hb low Ferritin	5.8	0.5	0.000	5.0	0.5	0.050
	IDA	4.9	0.2		4.7	0.5	
Hb	Normal Hb low Ferritin	13.3	0.3	0.000	12.6	0.6	0.000
	IDA	10.6	1.1		9.3	1.4	
MCV	Normal Hb low Ferritin	72.1	1.5	0.758	81.3	5.2	0.000
	IDA	71.2	6.3		68.1	7.5	
MCH	Normal Hb low Ferritin	23.1	1.3	0.139	25.4	2.2	0.000
	IDA	21.5	2.2		19.9	3.2	
MCHC	Normal Hb low Ferritin	32.0	1.1	0.039	31.2	1.0	0.000
	IDA	30.2	1.8		29.0	1.9	
RDW	Normal Hb low Ferritin	15.3	2.0	0.305	14.8	1.8	0.000
	IDA	16.3	2.0		19.2	3.1	
PLT	Normal Hb low Ferritin	410.0	37.2	0.920	286.2	85.0	0.024
	IDA	416.8	159.1		361.9	109.4	
Iron	Normal Hb low Ferritin	54.2	17.7	0.001	60.4	32.1	0.000
	IDA	26.8	12.2		28.7	15.8	
UIBC	Normal Hb low Ferritin	521.4	257.0	0.054	353.0	101.5	0.028
	IDA	364.5	55.1		406.7	70.2	
Ferritin	Normal Hb low Ferritin	19.2	4.5	0.198	13.1	6.7	0.000
	IDA	13.3	10.1		6.3	5.2	
RC	Normal Hb low Ferritin	0.8	0.3	0.568	1.1	0.3	0.426
	IDA	0.9	0.3		1.2	0.4	
Ret-Hb	Normal Hb low Ferritin	25.8	1.2	0.001	27.1	2.9	0.000
	IDA	21.7	2.1		19.1	4.5	

**Table 4:** Correlation of reticulocyte hemoglobin content with other parameters among IDA patients.

		HGB	RC%	Iron	Ferritin
Ret-Hb	r	0.819	0.070	0.232	0.540
	*p*	0.000	0.585	0.065	0.000
